# Uncommon Complication Post-deep Sclerectomy: Giant Retinal Tear

**DOI:** 10.7759/cureus.53854

**Published:** 2024-02-08

**Authors:** Mohammed N Felemban, Merai Alshehri, Faisal F Aljahdali, Marcos Rubio, Konrad Schargel

**Affiliations:** 1 Ophthalmology, Heraa General Hospital, Makkah, SAU; 2 Surgery, College of Medicine, University of Bisha, Bisha, SAU; 3 College of Medicine, King Saud bin Abdulaziz University for Health Sciences, Jeddah, SAU; 4 Ophthalmology, King Khaled Eye Specialist Hospital, Riyadh, SAU

**Keywords:** congenital glaucoma, open angle glaucoma, rhegmatogenous retinal detachment, retinal tear, deep sclerectomy

## Abstract

Glaucoma is a prevalent neurodegenerative disease. It causes progressive visual loss and is one of the most common causes of blindness worldwide. It can be categorized into open-angle or closed-angle glaucoma. Primary congenital glaucoma (PCG) is a subdivision of open-angle glaucoma. Non-penetrating deep sclerectomy (NPDS) is a surgical method for managing open-angle and primary congenital glaucoma, which was first introduced in 1990. During NPDS, a sclera flap is raised but not completely removed, and the outer part of Schlemm's canal and trabecular meshwork, along with the juxtacanalicular tissue, are excised without completely penetrating the eye. Therefore, it is considered a safe and efficient option for controlling intraocular pressure. This report shows a unique case of uncommon complication post-deep sclerectomy, a giant retinal tear, after undergoing non-penetrating deep sclerectomy for primary congenital glaucoma.

## Introduction

Glaucoma is a common neurodegenerative disease of the eyes that causes progressive visual loss and may lead to blindness. It can be divided into open- or closed-angle glaucoma. A form of open-angle glaucoma is primary congenital glaucoma (PCG). The primary management option for refractory PCG is always surgical. Goniotomy, trabeculotomy, trabeculectomy, and non-penetrating deep sclerectomy (NPDS) can all be options for treating primary congenital glaucoma [1].

NPDS is one of the procedures used to manage primary congenital glaucomas. Multiple reports have shown its superior safety to conventional filtering glaucoma surgeries [2-4]. NPDS allows aqueous filtration from the anterior chamber to the subconjunctival space through a thin trabeculodescemetic membrane, avoiding the sudden intraocular pressure (IOP) drop and thus lowering the incidence of post-operative complications associated with trabeculectomy and glaucoma tube surgeries [5,6]. For example, lens injury, inflammation, iris trauma, sudden hypotony, choroidal detachment, flat anterior chamber, hyphema, cataract, and endophthalmitis are all possible complications that may happen with penetrating surgeries [6-8].

Uncommon complications that may happen with NPDS are Descemet membrane detachment (DMD), hypotony, and hyphema [9], and ocular decompression retinopathy [10]. In addition, rhegmatogenous retinal detachment (RRD) with a giant retinal tear is considered a rare complication following post-NPDS. The present report shows a unique case of uncommon complication post-NPDS, an RRD with a giant retinal tear, after undergoing NPDS for primary congenital glaucoma [10].

## Case presentation

A 16-year-old male, who was a known case of primary congenital glaucoma in both eyes and anisometropia amblyopia of the right eye, was presented to our hospital. The patient was treated medically with brinzolamide plus timolol in a fixed combination (Azarga) and bimatoprost (Lumigan) in the right eye and bimatoprost (Lumigan) in the left eye, and he presented to the emergency room (ER) in King Khaled Eye Specialist Hospital with high, uncontrolled intraocular pressure in the right eye.

Clinical examination showed no dysmorphic features, but right megalocornea and buphthalmos were noticed. Right eye (OD) examination revealed corneal Haab's striae, a deep anterior chamber, and a normal lens. Funduscopic examination confirmed optic disc cupping (cup-to-disc ratio: 0.95 with inferior thinning) and myopic fundus without any sign of ocular inflammation, vitreous or retinal hemorrhages, breaks, or detachment. Left eye (OS) examination revealed a clear cornea, a deep anterior chamber, and a normal lens. Funduscopic examination showed an optic disc cupping (cup-to-disc ratio: 0.6). IOP on arrival in ER was as follows: OD 45 mmHg, OS 22 mmHg, and post-STAT antiglaucoma medication (brimonidine 0.15% drop/timolol maleate - 0.5% every three times with 10 minutes apart plus acetazolamide 250 mg two tablets) the readings were as follows: OD 30 mmHg, OS 15 mmHg. Visual acuity OD 20/100 improved by pinhole to 20/60 OS 20/20. The pachymetry showed central corneal thickness of 478 μm OD and 495 μm OS.

The patient was admitted and medical management started as follows: apraclonidine (Iopidine) 0.5%, brinzolamide plus timolol in a fixed combination (Azarga), and bimatoprost (Lumigan) in the right eye, but the IOP was still above target and booked for surgical control of the IOP; NPDS in the right eye under general anesthesia performed as below.

The patient was prepared and draped in the usual aseptic manner for a sterile ophthalmic procedure. A wire speculum was applied to open the lids. A clear corneal traction suture of #7-0 Vicryl was placed from 11 o'clock to 1 o'clock, and the eye rotated inferiorly. A fornix-based conjunctival flap was made between 10 o'clock and 2 o’clock. Hemostasis was achieved with light cautery. A trapezoidal marker for partial-thickness superficial scleral flap was used, 5 mm (anterior border) × 5 mm (long) × 4 mm (posterior border), and was dissected with a MANI blade. Filter papers soaked in mitomycin C solution 0.02% for 2 minutes were applied under the conjunctiva far posterior (Kwan technique) and under the scleral flap, after which the filter papers were removed, and the field irrigated generously with 100 mL balanced salt solution. A trapezoidal marker for the second scleral flap was also used, 4 mm (anterior border) × 4 mm (long) × 3 mm (posterior border), and was also dissected with a MANI blade to almost full-thickness under the first flap and into the clear cornea, making a wide trabeculo-Descemet window. We made a paracentesis and decompressed the eye carefully; then, we injected Miochol intraocular. The remanent scleral tissue from the second flap was excised with special deep sclerectomy scissors. The inner wall of Schlemm’s canal was scraped and peeled (with the Mermoud scraper and Mermoud forceps). Also, the Schlemm's ostium was checked with the Mermoud spatula. After checking for adequate filtration, right outflow, and anterior chamber stability, the first scleral flap was closed by apposition; no suture was used. The conjunctiva was closed using #9-0 Vicryl suture. Subconjunctival injections of cefazolin 50 mg and dexamethasone 2 mg were given. The lid speculum and traction suture were removed. In the end, topical medications of Maxitrol ointment and pilocarpine, 2% drops, were applied, followed by a patch and shield.

Post-operative treatment included antibiotic moxifloxacin (Vigamox), and steroid prednisolone (Optipred) eye drops four times a day for one week, followed by gradual reduction of the steroids over four weeks and two weeks of the antibiotic course. On day one post-operatively, the IOP was 7 mmHg OD. The patient was discharged with a stable condition and unremarkable B-scan. One week post-operatively, the IOP was 18 mmHg OD. The anterior chamber and the bleb are well-formed in the right eye. Timolol was started and a follow-up appointment was given.

**Figure 1:**
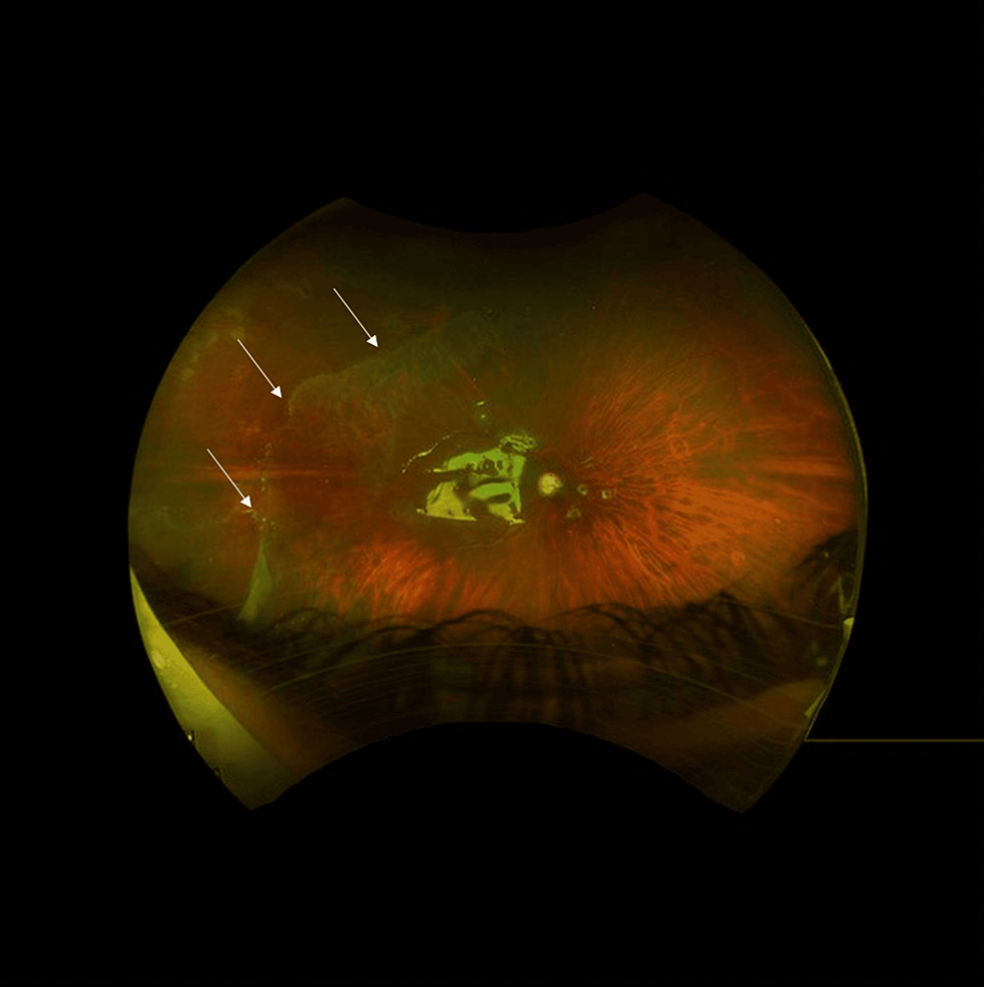
Post-operative day one retinal flat under silicone oil, small temporal fold. The white arrows are pointed on the small temporal fold.

After two weeks, the patient returned to the emergency room complaining of sudden vision loss for five days. RRD was diagnosed with a giant retinal tear 160 degrees involving the macula in the right eye. The patient was admitted, and retina surgery was performed. Pars plana vitrectomy with silicone oil tamponade 1300 CS plus endolaser over the tear's posterior border and 360 on the periphery right eye under general anesthesia. The next day the retina was flat under oil with good laser, small fold temporally (Figure 1).

The patient was discharged in good general condition. Ten days after that, the patient came for a follow-up and the examination revealed the following findings: visual acuity=OD: 20/200, OS: 20/20, and IOP measured by iCare=OD: 16 mmHg, OS: 16 mmHg.

## Discussion

Glaucoma refers to a collection of optic neuropathies that involve the gradual deterioration of retinal ganglion cells. These cells are a type of neuron found in the inner retina, with axons extending into the optic nerve. As these nerves degenerate, the result is a characteristic appearance of the optic disc known as cupping, which is accompanied by a loss of vision. Despite ongoing research, the underlying biological mechanisms of glaucoma are not yet well understood, and the factors contributing to its progression are not fully defined [11]. It can be classified into the following two main types: open-angle and closed-angle. Primary congenital glaucoma is a type of open-angle glaucoma. Goniotomy, trabeculotomy, trabeculectomy, and NPDS can all be options for treating primary congenital glaucoma [5].

NPDS was initially introduced in 1990, the purpose of this technique was to prevent the serious potential complications associated with penetrating surgeries, such as low eye pressure, bleeding within the eye, a flattened anterior chamber, detachment of the choroid, fluid buildup or bleeding, and inflammation within the eye [12]. They described the surgical procedure that involved removing a piece of the innermost layer of the eyeball, exposing Schlemm's canal, and removing a layer of tissue beneath it. This created a space in the sclera, allowing aqueous humor to drain. The removal of this tissue specifically targeted the area of the eye with the greatest resistance to fluid drainage, resulting in increased drainage and decreased eye pressure [6]. However, there are a few complications that may happen post-NPDS. For example, hypotony is one of the most common and early complications after NPDS. Other complications associated with hypotony are choroidal detachment, retinal detachment, shallow AC, and hypotonous maculopathy, but these complications are rare [12].

RRD is a medical condition characterized by the separation of the neurosensory retina from the retinal pigment epithelium due to a break in the retina. RRD is the most prevalent form of retinal detachment (RD) and can significantly impair a patient's vision. Therefore, if not treated urgently, it may lead to blindness [2]. RD after NPDS has been reported in the literature only in patients with Sturge-Weber syndrome. Taherian and Anand reported the coexistence of peripapillary retinoschisis and exudative RD after NPDS [13,14]. With aging, collagen in the vitreous hardens, and elasticity decreases, separating the vitreous from the retina [10]. As a result, possible holes in the retina may form where the vitreous is still attached to the retina, excreting its most traction force; however, our patient in the present case is young [11]. RRD is more after penetrating and combined surgeries; however, our patient has undergone single non-penetrating surgery. Local anastatic block can cause globe perforation with RRD [15]. However, our patient underwent general anesthesia. On the other hand, myopia is more prevalent in children with congenital glaucoma, as in our case. The risk of RD in myopic patients with >-3 diopters increases 10 times more than in the normal population. A possible cause is the early liquefaction of the vitreous in the myopic eye [3,4]. Regular meticulous post-operative follow-up with complete ocular examination, including fundus examination, is recommended to avoid missing such serious rare complications.

## Conclusions

Glaucoma is a disease that causes irreversible visual loss. It is divided into open- or closed-angle glaucoma. PCG is a type of open-angle glaucoma. The definitive management of PCG is mainly surgical. NPDS is considered an efficient surgical option for PCG due to its safety compared to other filtering surgeries. However, a good examination from the periphery of the retina by direct observation or B-scan should probably be done as a routine to discard atrophies, holes, or other anatomical malformations of the retina and be able to treat it. Even knowing this will not be a warranty, totally avoid RD.
